# Suicide prevention strategies for older persons—An integrative review of empirical and theoretical papers

**DOI:** 10.1002/nop2.789

**Published:** 2021-02-23

**Authors:** Anne Lise Holm, Elin Salemonsen, Elisabeth Severinsson

**Affiliations:** ^1^ Faculty of Health and Social Sciences Western Norway University of Applied Sciences Haugesund Norway; ^2^ Nursing and Healthcare Research Group Department of Research Stavanger University Hospital Stavanger Norway

**Keywords:** communication, integrative review, older adults, prevention, social support, suicide

## Abstract

**Aim:**

To synthesize suicide prevention strategies for older adults. The review question was Which suicide prevention strategies are useful for older adults?

**Design:**

Integrative review.

**Data sources:**

Academic Search Premier, CINAHL, Ovid PsycINFO and PubMed were searched for articles published between January 2009 and December 2019.

**Review methods:**

An integrative review of quantitative, qualitative and theoretical papers with a qualitative thematic analysis.

**Results:**

Key aspects of the included studies contributed to the formulation of four themes: (1) Recognizing older adults’ physical and/or mental health problems and referring them for help and treatment, (2) Designing an educational programme, (3) Communication and dialogue about warning signs and (4) Social support and awareness of causing significant others emotional pain. The findings indicate an urgent need to identify effective suicide prevention strategies for older adults.

## 1 INTRODUCTION

The statistics on suicide among older adults are alarming (Greenlee & Hyde, 2014 and the rate is far greater than that of younger people (World Health Organization (WHO), 2014). In the United States, older adults complete suicide at a rate of 16.1 (per 100,000 people). This is higher than the national rate of 13.0 and the rate for young people (11.1) (WHO, 2014). Moreover, older adults suffer from more chronic health problems that can reduce their quality of life (Greenlee & Hyde, 2014). According to Winterrowd et al. (2017), health problems were perceived as more acceptable precipitants for suicide by older adults compared with social or mental issues. A gender difference has been found in the suicide rate for men aged over 60 years (Oyama et al., 2010). The social context is an important factor for understanding suicide (Hjelmeland, 2011) and making sense of the overwhelmingly high suicide rates in older men, especially in the United States, Canada and many European countries (Fässberg et al., 2012).

Mental illness is described as a risk factor that contributes to suicide in older adults. The level of mental illness in older adults is even higher than that found among younger people (De Leo et al., 2004; Lapierre et al., 2011). Butcher and Ingram (2018) described mental illnesses such as borderline personality disorder and anxiety disorders as risk factors for suicide. According to Butcher and Ingram (2018, p. 22), ‘some older adults experience so much physical and emotional pain that they feel hopeless about being able to change and improve their life’. Depression is described as a risk factor for suicide, especially in older women (Butcher & Ingram, 2018; Crump et al., 2013; Unützer, 2007), while the treatment rate for depression among older men is low (Hinton et al., 2006). Despite the fact that older adults who die by suicide often visit healthcare centres due to depression, the risk of suicide is not addressed (Ahmedani et al., 2014; Crump et al., 2013). Depression is associated with conditions such as **‘**arthritis, cardiovascular disease and cancer’ that often affect older adults (Greenlee & Hyde, 2014, p., 23). In addition, it has been found to be a side effect of different medications such as statins, beta‐blockers and corticosteroids (Greenlee & Hyde, 2014; Neel, 2012). Depression is mentioned in studies of older adults suffering from early‐stage dementia (Draper et al., 2010; Fässberg et al., 2012; Kim & Hyun, 2013) and has been described as a risk factor in two systematic reviews of older suicidal adults (Holm & Severinsson, 2015; Okolie et al., 2017).

Systematic reviews and reviews worldwide indicate the importance of different suicidal prevention strategies for adolescents and adults. A review of systematic reviews by Van der Feltz‐Cornelis (2011) identifies best practice multilevel suicide prevention strategies such as training general practitioners to recognize and treat depression and suicidality, improving accessibility of care for at‐risk people and restricting access to means of suicide. The survey by Claassen et al. (2014) aimed at reducing the burden of suicide in the United States suggested several suicidal prevention strategies including retaining patients in care, improved healthcare provider training and generating care models to ensure accessible treatment (Claassen et al., 2014). A review of 10‐year systematic reviews from 2005–2014 (Zalsman et al., 2016) found evidence for restricting access to lethal means by improved control of analgesics and hot‐spots for suicide by jumping; school‐based awareness programmes; and pharmacological and psychological treatments of depression (Zalsman et al., 2016). A review from Australia by Nicholas et al. (2018) suggested a suicide prevention media campaign indicated that family members or friends should ask directly about suicidal thoughts and intentions. They should listen to the responses without judgment and tell the person at risk that they care and want to help (Nicholas et al., 2018). A review by Oexle et al. (2019) suggested suicide prevention strategies such as scanning social media posts, prediction models using electronic health record data as a way to enhance the suicidal process (Oexle et al., 2019). A recent systematic review by Wolitzky‐Taylor et al. (2020) from the United States found significant increases in suicide prevention knowledge, skills and self‐efficacy in the prevention of suicide. The strategies employed gatekeeper training. Evidence of reductions in suicidal ideation and behaviours was observed in the suicide prevention programmes (Wolitzky‐Taylor et al., 2020). A study by Page et al. (2017) developed a decision‐support tool to prevent suicide in Australia. Findings suggested that the largest reductions in suicide (12%) were associated with general practitioner training and coordinated aftercare approaches (Page et al., 2017). A theoretical study by Mokkenstorm et al. (2018) was based on the implementation of a suicide protection guidelines in specialist mental healthcare institutions in the Netherlands. The guidelines significantly improved the development of an organizational suicide prevention policy; monitoring and trend‐analysis of suicide numbers; evaluations after suicide; and clinician training (Mokkenstorm et al., 2018). A study from Japan (Nakanishi et al., 2020) also implemented a suicide prevention act, where 597,007 suicides were analysed. However, the suicide prevention act was not significant overall or for any stratified populations (Nakanishi et al., 2020).

Systematic, integrative or scoping reviews of effective suicidal prevention strategies for older adults are scarce. One systematic review by Lapierre et al. (2011, p. 88) illuminated a suicide prevention programme for older adults and stated that ‘most studies were centred on the reduction of risk factors such as depression treatment, and decreasing isolation, but when gender was considered the programs were mainly effective for women’. One theoretical study by Sakashita and Oyama (2019) described linkages between universal, selective and indicated prevention strategies. Universal prevention strategies included mental health policies, awareness‐raising, education, improved access to health care and population or universal screening. Selective prevention interventions included gatekeeper training for physicians, robust screening and counselling of at‐risk individuals, the availability of crisis helplines and interventions for vulnerable people (those experiencing severe stress). Indicated prevention strategies included assessment and management of mental disorders associated with suicidal behaviours, community support and psychosocial follow‐up (Sakashita & Oyama, 2019).

The reason for performing this integrative review is the lack of research on suicide prevention strategies for older adults. Only one systematic review of a suicide prevention programme for older adults was found (Lapierre et al., 2011). The rationale behind this integrative review is that the authors did not find any systematic, integrative, or scoping review study from 2009–2019 that illuminated suicide prevention strategies for older adults.

## 2 AIMS

The aim of the review was to synthesize suicide prevention strategies for older adults. The review question (RQ) was Which suicide prevention strategies are useful for older adults?

## 3 METHODS

The integrative method includes both empirical and theoretical publications (Evans, 2007).

### 3.1 Design

An integrative literature review was used, in which the researchers summarize and make conclusions about a subject (LoBiondo‐Wood & Haber, 2010). In addition, we conducted a systematic search, an evaluation of quality criteria as well as a thematic synthesis or analysis of past qualitative and quantitative studies (LoBiondo‐Wood & Haber, 2010). The risk of the reviewer being overwhelmed by the volume of literature is therefore greater during an integrative review, as a large number of irrelevant studies have to be screened to identify the few that are relevant.

**FIGURE 1:**
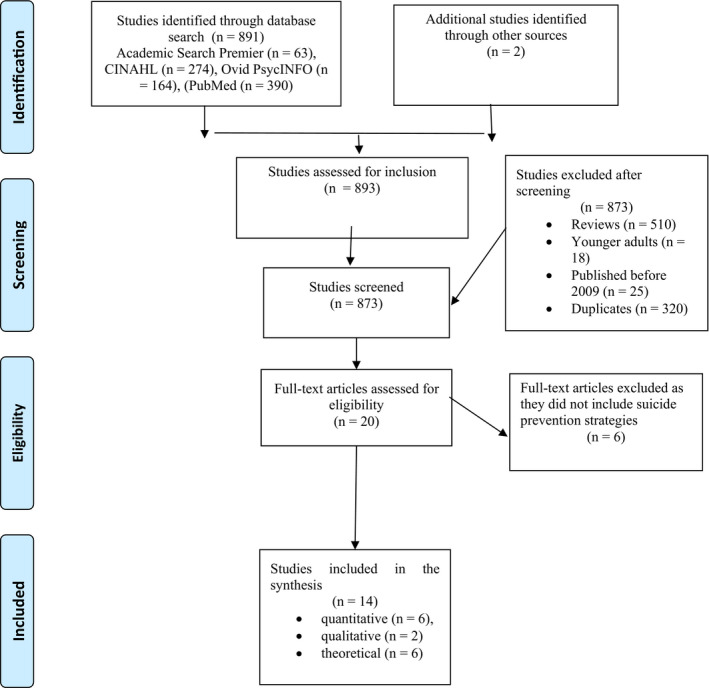
Appendix I: PRISMA 2009 flow diagram. From: Moher et al. (2009). For more information, visit www.prisma‐statement.org

Whittemore and Knafl (2005) included five stages that guided the integrative review design. The first stage was problem identification including the research question and aim. The second stage comprised a literature search involving a comprehensive search strategy (Figure 1). The third stage involved data evaluation, where the three authors evaluated the methodological quality of the included studies (Table 1, Table S1, Hong et al., 2018; Lotfi et al., 2019). In the fourth stage, which concerned data analysis, data on sample characteristics and methods as well as references to the concept of integration were extracted from primary sources (Whittemore, 2005). Related terms and relationships to integration were identified. A data matrix was developed to present the data from each report with the help of suitable themes from the material. The three authors read the included articles and conducted a data analysis, mainly on a manifest level. In the fifth data presentation stage, a synthesis of the findings was developed to illustrate the process of integration based on an example from Whittemore (2005).

**TABLE 1:** Methodological characteristics of the included quantitative (*n* = 6) and qualitative (*n* = 2) studies

	Researchers & Date/Country	Study design	Study sample	Measurement	Data analysis	Limitations
	Quantitative studies					
1	Chan et al. (2011), CHINA	Cohort design.	Pre‐intervention group (*n* = 66) and Intervention group (*n* = 351).	Hong Kong Government mortality statistics (Census and Statistics Department).	SPSS, 17.0	Use of a historical cohort does not permit adequate control for characteristics as in randomized controlled trials. Uncontrolled baseline factors might partly account for the difference in the 2‐year suicide rates.
2	Chan et al. (2018), CHINA	Explorative design.	Community dwelling older adults, *n* = 106,583,	Self‐reported questionnaires. Data from the Coroner's Court, which holds the official suicide statistics for Hong Kong.	Secondary data analysis	The study is a retrospective secondary data analysis.
3	Chauliac et al. (2016), FRANCE	Quasi‐experimental design.	12 nursing homes where 30% of the staff had undergone gatekeeper training and 12 nursing homes without trained staff.	The intervention consisted of two days of training, a total of 14 hr, attended by at least 30% of the nursing home staff, including support staff.	Software package, Statistical Analysis System, SAS.	There are two main limitations. One is that the researchers could not randomly choose the nursing homes. The second is that we were unable to establish a baseline for judgment criteria before the study started. This might have led to unmeasured original differences between the two groups.
4	Ho et al. (2014), SINGAPORE	Case–control design.	Older adults (*n* = 409) who died by suicide in Singapore between 2000–2004.	Singapore Registry of Births and Deaths between 2000–2004.	Statistical Analysis System, SAS	This retrospective register‐based study had reporting bias, recall bias, limited information and no examination of causality. Recall bias among informants is inevitable. The study cannot be generalized to other populations.
5	Kim and Yang (2017), SOUTH KOREA	Quasi‐experimental design.	Community dwelling older adults (*n* = 62). One experimental group (*n* = 31) and one control group (*n* = 31).	Mini‐Mental State Examination (MMSE), the Consortium to Establish a Registry for Alzheimer's (CERAD) inventory (MMSE‐KC). The Suicidal Ideation Scale (SIS). Perceived health status, Activities of daily living, Social support, The Geriatric Depression Scale Short Form‐Korean version (GDSSF‐K).	SPSS (ver.21.0, SPSS Inc).	Not mentioned
6	Karakus et al. (2015), TURKEY	Descriptive design.	Community dwelling older adults (*n* = 150), (*n* = 95 male), (*n* = 55 female).	Geriatric autopsies were evaluated retrospectively between January and December 2006.	Chi‐squarePearson and Fisher exact test.	Not Mentioned
	Qualitative studies					
1	Vannoy et al. (2018), USA	Qualitative design.	Community dwelling older men (*n* = 77).	Interviews.	Thematic analysis.	The sample consisted of older, low‐income Mexican‐origin and white men utilizing health services, thus cannot necessarily be generalized to those not utilizing health services. The study focused on men from low‐income backgrounds. Older men from other backgrounds might have very different views on this topic. The results should be considered preliminary rather than definitive. The team included a range of clinical and theoretical perspectives and most have been involved in research and practice innovation related to improving mental health services in primary care settings.
2	Wong et. al. (2011), CHINA	Qualitative case design.	Older adults at high risk of suicide (*n* = 8) were assessed and treated from 2002–2010.	8 cases.	Not mentioned	Mini‐Mental Stat**e** Examination (MMSE) score and Geriatric Depression Score (GDS) were used to screen for depressive symptoms. Only patients who had committed suicide while under the active care of the Elderly Suicide Prevention Program (ESPP) were included. No suitable controls were available to elucidate the differences in risk factors that could explain the differences in clinical outcomes. The cases were taken from one cluster, so the findings may not be generalized to elsewhere in Hong Kong.

### 3.2 Search strategy

The study was conducted by performing a search in Academic Search Premier, CINAHL, OVID PsycINFO and PubMed from December 2018 to October 2019. In Academic Search Premier, the authors used search words such as suicide prevention AND older adults. The inclusion criteria were as follows: peer‐reviewed, English language articles, which resulted in 63 articles (*n* = 63). In CINAHL, the search words were suicide prevention and the inclusion criterion was as follows: aged 65 + years, resulting in 274 articles (*n* = 274). In OVID PsycINFO, the search word was as follows: suicide prevention and the inclusion criteria were as follows: peer‐reviewed articles, English language, aged 65 + years and older. The result was 164 articles (*n* = 164). In PubMed, the search word was as follows: suicide prevention and the inclusion criteria were published in the last 10 years, aged 65 + years. The result was 390 articles (*n* = 390). Please see Figure 1 for more information about the search strategy.

### 3.3 Inclusion and exclusion criteria

The inclusion criteria were suicide prevention and older adults. We searched for studies that included ‘suicide prevention and older adults’ in the title to ensure that suicide prevention for older adults was the aim of the study. One empirical study where the median age was 64 years was included (Vannoy et al., 2018). An additional manual search took place in October 2019, which resulted in two studies. We identified a total of 893 studies and 873 were excluded due to being duplicates, review studies, focused on younger adults, published before 2009 and because the title did not include ‘suicide prevention’ and ‘older adults’. The remaining 20 studies were assessed for eligibility by reading the full text and of these six were excluded due to not including suicide prevention strategies (Figure 1). Hence, 14 studies remained; six quantitative, two qualitative and six with a theoretical approach.

### 3.4 Data synthesis

In this integrative review, suitable themes from the literature were identified and the findings synthesized under thematic headings (Evans, 2007). The papers were read several times and verified by the three authors. The five stages recommended by Whittemore and Knafl (2005) were included in this process, which was time consuming due to the intention of selecting parts related to different stages of the data synthesis process. Information related to the aim was written down and read several times. Relevant descriptions were coded and categorized into preliminary themes. Finally, the first author (ALH) ensured that the papers were related to the methodological characteristics and quality criteria (Table 1) and that the results matched the present study.

## 4 RESULTS

### 4.1 Methodological characteristics and quality criteria of the included empirical studies

According to Whittemore and Knafl (2005), evaluating quality criteria by means of an integrative review method increases the complexity. We have described the quality criteria of the included empirical studies in Table 1 and Table S1.

This integrative review included six quantitative and two qualitative studies with different designs. These empirical studies outlined their designs as cohort (1), quasi‐experimental (2), case–control (1), explorative (1), descriptive (1), qualitative (2) and theoretical (6). The six theoretical studies are outlined in Table 3.

One of the quantitative studies was described as having an observational cohort design (Chan et al., 2011). Observational (non‐experimental) designs are sometimes used when a researcher wants ‘to construct a picture of a phenomenon or explore events, people or situations as they naturally occur in the environment’ (Schneider et al., 2007, p 0.159). The included cohort design study comprised data from the elderly suicide prevention program (ESPP) from 2002–2007 (Chan et al., 2011). Old data can be a limitation that can threaten both validity and reliability in addition to distorting the results (Polit & Beck, 2017). According to Polit and Beck (2017), this can be related to the time factor and to internal validity, which means that the data may have changed since data collection.

Two quantitative studies had explorative (Chan et al., 2018) and descriptive designs (Karakus et al., 2015). Exploratory and descriptive studies have an observational design that often uses a questionnaire or interview to collect data from individuals, groups and institutions (Schneider et al., 2007). The ability of such non‐experimental studies to support causal inferences is weak. In addition, the study by Chan et al. (2018) was retrospective and based on a secondary data analysis. There was no description of recall bias that is associated with a retrospective design. The quantitative study by Ho et al. (2014) was also retrospective as well as being a case–control study. Ho et al. (2014) stated that the study could increase recall bias and other limitations mentioned were sparse information and no examination of causality. Self‐reports gather retrospective data about events that occurred in the past or information about behaviours in which people plan to engage in the future. Self‐reports can be seen as a retrospective data collection method and thus increases bias due to the informants’ inability to remember what exactly happened or what they experienced in the past (Polit & Beck, 2017). Polit and Beck (2017) highlights that self‐reports can reduce the validity of the study. According to Holm et al. (2019, p. 13), ‘self‐reports can also be related to unmeasured confounders’.

Two quantitative studies employed a quasi‐experimental interventional design (Chauliac et al., 2016; Kim & Yang, 2017). In quasi‐experimental designs, the researcher manipulates experimental treatment but some characteristics of a ‘true’ experiment are lacking—either control or randomization (Schneider et al., 2007, p. 163, 164). Schneider et al. (2007, p. 164) explain that this design can threaten ‘a study's internal validity’ and weaken any causal inference. The sample in both studies was small (Table 1, S1), which can increase bias.

Four of the quantitative studies described methodological limitations (Chan et al., 2011, 2018; Chauliac et al., 2016; Ho et al., 2014) (Table 1). Two of the studies mentioned using a smaller sample size that can increase bias (Chauliac et al., 2016; Kim & Yang, 2017) (Table 1).

Two qualitative studies were included (Vannoy et al., 2018; Wong et al., 2014). Neither contained any reflections or limitations related to the use of the qualitative method.

Demographic characteristics in the quantitative and qualitative studies (*n* = 8) are presented in Table 2. One study contains no information about the health status of the participants (Chauliac et al., 2016). One study has no information about the participants’ living situation (Karakus et al., 2015), although five studies contain such information (Table 2).

**TABLE 2:** Demographic characteristics of the included quantitative (*n* = 6) and qualitative (*n* = 2) studies

	Researchers	Ethnicity	Age and sex	Health status	Living situation	Financial status
	Quantitative studies					
1	Chan et al. (2011), CHINA	Hong Kong Chinese	Pre‐intervention, 36.4% male, 63.6% female. Postintervention ESPP group: 42.7% male, 57.3% female. 65 years and older.	In the pre‐intervention group, 6.67% had a diagnosis of depressive disorder. In the postintervention, 2.35% had a diagnosis of depressive disorder.	6.67% lived alone in the pre‐intervention group; 6.45% lived alone in the post intervention group.	In the pre‐intervention group, 40.9% received support from the social security system. In the ESPP group, 34.2% received such financial support.
2	Chan et al. (2018), CHINA	Hong Kong Chinese	34.3% male, 65.6% female. 65 years and older.	Cardiovascular diseases 73.6%, Eye disease 39.2%, Skeletal system diseases 26.9%, Endocrine system diseases 26.7%, Brain and nervous system diseases 15.4%, Respiratory diseases 10.7%, Mental Illnesses, 2.5%.	40.8% lived alone.	41.6% were social welfare recipients.
3	Chauliac et al. (2016), FRANCE	NM	26.8% male, 73.2% female. Age NM	NM	Living in nursing homes.	100% were social welfare recipients.
4	Ho et al. (2014), SINGAPORE	49.7% were born in Singapore, while 50.3% were born in other countries.	61.1% male, 38.9% female. Mean age 73.7.	Physical illnesses 70.9%, Schizophrenia, major depressive disorder, bipolar disorder 12.7%. Anxiety, minor depressive disorder, adjustments disorder, acute stress reaction 35.2’%. Personality disorder 12.2%. Alcohol misuse 2%.	25.2% lived alone, 6.1% single, 74.6% married, 19.1% divorced or widowed.	NM
5	Kim and Yang (2017), SOUTH KOREA	NM	29% male, 81% female. 65 years and older.	Alzheimer 45.2%, Alzheimer, 54.8% Vascular dementia.	19.4% lived alone, 29% were living with a spouse and 51.5% were living with children.	Low economic status 35.4%, Moderate 48.4%, High 16.2%.
6	Karakus et al. (2015), TURKEY	NM	63.3% male, 36.7% female. Mean age males 73.3, mean age females 76.2.	NM	NM	NM
	Qualitative studies					
1	Vannoy et al. (2018), USA	Non‐Hispanic white, 61%, Mexican origin, 39%.	100% male. Mean age 64 years.	Past‐year clinical depressive disorder 78%. No past‐year clinical depressive disorder 22%.	60% married, 40% divorced, separated, widowed, cohabitating.	Under $10,000 28%, $10,000‐$25,000 38%, $25,000‐$50,000 17%, $50,000‐$75,000 4%, $75,000‐$100,000 8%, over $100,000 1%.
2	Wong et al. (2011), CHINA	NM	62% male, 37% female.	100% depressive disorder.	50% lived alone, 50% lived with family.	NM

Note

NM, not mentioned, NR, not relevant.

### 4.2 Summary of the characteristics of the included papers

Key aspects of the included studies contributed to the synthesis of the empirical and theoretical papers, revealing four themes and one sub‐theme. The first theme: Recognizing older adults’ physical and/or mental health problems and referring them for help and treatment was based on the sub‐theme: Training gatekeepers or para professionals. The second theme was Designing an educational programme, while the third was Communication and dialogue about warning signs and the fourth was Social support and awareness of causing significant others emotional pain.

#### 4.2.1 Recognizing older adults’ physical and/or mental health problems and referring them for help and treatment

Three quantitative (Chan et al., 2018; Ho et al., 2014; Kim & Yang, 2017), two qualitative (Vannoy et al., 2018; Wong et al., 2014) (Table 2) and one theoretical study (Arbore, 2019) revealed that older adults who died by suicide had a past history of suicidal behaviour and suffered from mental illness.

Ho et al. (2014) found that having received mental health treatment in the past and having antidepressant medication detected by blood toxicology screening were indicators of suicide ideation. Those patients without suicidal behaviour often have a suicide plan in addition to having received medical or surgical treatment in the past (Ho et al., 2014). Kim and Yang (2017) suggest using a community‐based programme for healthcare professionals to prevent suicide among older adults with early‐stage dementia.

Vannoy et al. (2018) found that the participants struggled with inner conflicts that involved their identity and self‐image. Suicidal ideation violate**s** their self‐image and what other people think and expect of them (Vannoy et al., 2018). Wong et al. (2014) revealed that the participants (older men) had been admitted to either a mental or general hospital about 1 month before committing suicide and were diagnosed with a depressive illness.

The theoretical study by Arbore (2019) highlighted the importance of responding to the needs of people who are experiencing emotional pain.

##### Training gatekeepers or paraprofessionals

Two quantitative studies (Chan et al., 2011; Chauliac et al., 2016) and three of the theoretical studies (Arbore, 2019; Conwell, 2014; Fullen, 2016) suggested training gatekeepers to identify those in need of help and treatment.

Chan et al. (2011) found that involving a gatekeeper in primary care such as physicians, social workers, healthcare professionals, and volunteers and others non‐specialists in psychiatric care. These volunteers can be helpful for identifying a person at risk of suicide and making an urgent referral. It can be useful to assign a psychogeriatric nurse in a psychiatric institution as a coordinator who can arrange an appointment within a short time. Chauliac et al. (2016) found that when a suicidal person was identified, one strategy was an appointment with a psychologist, or a general practitioner. Thus, trained healthcare professionals at the nursing home more often arranged an appointment with a psychologist (Chauliac et al., 2016).

The theoretical study by Arbore (2019) wanted to share responsibility for preventing suicide between (para) professional and lay members of the community. Conwell (2014) revealed that the lethal nature of suicidal behaviour indicated that more distal risk factors and approaches to prevention are necessary if reduction is to be achieved. Fullen (2016) suggested educating healthcare professionals and volunteers in the community in order to increase collaboration as a means of preventing suicide among older adults.

#### 4.2.2 Designing an educational programme

Two quantitative studies (Chan et al., 2011; Karakus et al., 2015) and one theoretical study (Fullen, 2016) recommended an education programme for preventing suicide in older adults. Chan et al. (2011) suggested that a care manager could provide psychoeducation in the first 6 months. Karakus et al. (2015) recommended an education programme for preventing suicide among older adults, which they assume will be necessary in 21st. century. The design of an education programme seems to have forced health educators within geriatric care to update their curriculum plans (Karakus et al., 2015).

The theoretical study by Fullen (2016) stated that educating healthcare professionals within different municipalities will be essential, especially in terms of how to look for warning signs and when to contact the healthcare services.

#### 4.2.3 Communication and dialogue about warning signs

One quantitative (Chan et al., 2018), one qualitative (Vannoy et al., 2018) and two theoretical studies (Butcher & Ingram, 2018; Van Orden & Deming, 2018) suggested that preventing suicide required communication and dialogue about warning signs.

Chan et al. (2018) described communication and decision‐making via a telecare helpline that was found helpful for preventing suicide. Vannoy et al. (2018) suggested some approaches for preventing suicide by older men, namely talking about depression, and about the impact of suicide on others. In addition, a certain attitude is necessary in order to discuss suicide with a healthcare professional (Vannoy et al., 2018).

The theoretical studies by Butcher and Ingram (2018) and Van Orden and Deming (2018) suggested that communication and dialogue must be related to making the family members look for warning signs and ask to talk to a professional or make an appointment with a care manager.

#### 4.2.4 Social support and awareness of causing significant others emotional pain

Two quantitative (Ho et al., 2014; Kim & Yang, 2017), two qualitative (Vannoy et al., 2018; Wong et al., 2014) and six of the theoretical studies (Arbore, 2018; Butcher & Ingram, 2018; Conwell, 2014; Demiricin et al., 2011; Fullen, 2016; Van Orden & Deming, 2018) revealed that social support and awareness of social roles can prevent suicide. The theoretical studies added that social connectedness, cohesion and pro‐social behaviour can reduce social isolation and loneliness (Arbore, 2019; Butcher & Ingram, 2018; Conwell, 2014; Demircin et al., 2011; Fullen, 2016; Van Orden & Deming, 2018).

Ho et al. (2014) suggested that psychiatrists should contact the social services in order to resolve the social problems experienced by older adults with a history of suicide ideation. Kim and Yang (2017) mentioned that protective factors in the suicide prevention programme were related to social support.

Vannoy et al. (2018) highlighted how suicide could impact on others in terms of pain and distress, particularly wives and children. Wong et al. (2014) suggested that professionals should pay special attention to festive periods such as holidays and Christmas. One of the participants committed suicide during the Christmas period.

The theoretical studies revealed the value of social networks and psychosocial treatment in addition to promoting connectedness in senior centres and senior residential communities (Arbore, 2019; Butcher & Ingram, 2018; Conwell, 2014; Demircin et al., 2011; Fullen, 2016; Van Orden & Deming, 2018). Arbore (2019) reported that rural suicide prevention efforts can be enhanced by collaboration between health and social service providers. Three theoretical studies pointed out that healthcare professionals must try to focus on social relationships and explore social isolation among older adults (Butcher & Ingram, 2018; Conwell, 2014; Fullen, 2016). Demircin et al. (2011) suggested that collaboration needs to be fostered across a broad spectrum of agencies and institutions in order to achieve this public health priority and reduce gaps in the understanding of issues associated with suicide prevention in old age (Demircin et al., 2011). Van Orden and Deming (2018) revealed the need to increase social engagement and suggested that such interventions must be implemented and available to all older adults.

## 5 DISCUSSION

Fourteen (eight empirical and six theoretical) papers were included in the final analysis. Key aspects of the included studies contributed to the formulation of four themes and one sub‐theme.

*Recognizing older adults’ physical and/or mental health problems and referring them for help and treatment*. In this integrative review, several studies revealed that suicidal older adults have poor health (Table 2). Earlier studies have highlighted physical health problems as risk factors for suicide among older adults (Preville et al., 2005). The illness‐suicide hypothesis has been questioned in relation to whether older adults who committed suicide had more chronic health problems than those who died of natural causes (Forma et al., 2017; Préville et al., 2005). Depression is cited as the most common reason for suicide among older adults (Chiu et al., 2004). Following a survey that screened for depression in older adults, the outcomes of a community‐based programme aimed at preventing were evaluated (Oyama et al., 2010). The risk for men was reduced by 61%, with a 51% risk reduction for women (Oyama et al., 2010). Depression in old age can last for many years, decreasing physical and social functioning, worsening chronic health problems and increasing mortality from suicide and other causes (Oakes et al., 2013; Unützer, 2007). For a long time, older men have had a high rate of completed suicide. Recognizing and treating depression and reducing access to firearms have been described as the most important actions that healthcare professionals can take to prevent suicide (Unützer, 2007). Draper (2014) highlighted that suicide prevention needs a lifetime perspective and that no single prevention strategy can be successful alone. Depression cannot be understood as something that is a normal part of the ageing process (Butcher & McGonigal‐Kenney, 2010; Butcher & Ingram, 2018). Healthcare professionals appear to believe that individuals are feeling sad due to their age, when in fact they have signs of depression (Centers for Disease Control, & Prevention, 2016; Dennis et al., 2007). Subsyndromal depression can be seen as existential depression and dispiritedness (Butler & McGonigal‐Kenney, 2010). Although existential depression does not meet the diagnostic criteria for major depressive disorder, it has often been related to various mental health issues, comorbidities, decreased functioning and quality of life, in addition to suicide risk (Butcher & Ingram, 2018; Pickett et al., 2014). Signs of existential depression can be a sense of meaninglessness, suicidal ideation and hopelessness. Several years ago, Shneidman (1993) described ‘psychache’ as the cause of suicidal thoughts and behaviour. Psychache was described as mental pain that overtakes the mind. This pain is localized within the brain in the Amygdala, the centre for emotions. Emotional pain is characterized by intense feelings of shame, guilt, fear, anxiety, or loneliness (Holm et al., 2014). Shneidman (1991, p. 40) stated that there is no difference between suicide in adolescents, adults or older adults; ‘there is only human suicide’ and all suicides must be understood in the same way. There is a worldwide increase in suicide by older adults, which is serious. Studies have revealed that mental health problems seem to be closely related to late‐life suicide, especially depression, bipolar affective disorder and anxiety disorders (Draper, 2014; Ladwig et al., 2010).

This integrative review revealed that *Training gatekeepers or paraprofessionals* had a significant effect on suicide prevention among older adults. Gatekeeper training can be a way that volunteers can assist healthcare professionals such as nurses, physicians, social workers and psychologists (Isaac et al., 2009). Gatekeepers can teach specific groups of people to identify suicidal adults and then refer them for treatment. Volunteers can also include different professional groups such as police, teachers and counsellors who have not been educated to intervene when someone is at risk of suicide. Family and friends are also suggested as potential gatekeepers based on their relationship with suicidal older adults (Isaac et al., 2009).

The findings suggest the need to give high priority to *Designing an educational programme* in order to prevent suicide in the community. Curriculum mapping as a tool for teaching and learning included different components so that the whole picture and the relationships and connections between the parts of the map are easily easy to see (Dent et al., 2013). This is valuable when developing suicide prevention strategies as shown in the present review. However, suicide prevention needs to reach into communities and cultures (Butcher & Ingram, 2018). It is important to address the negative attitudes related to ageism and develop educational strategies that can reduce discrimination of and prejudice towards older adults in general (Levy & McDonald, 2016) and suicidal older adults in particular.

*Communication and dialogue about warning signs* is one theme in the synthesis that is essential for suicide prevention. Older suicidal adults need someone to talk to when in a suicidal crisis. Communication via a telecare helpline can be helpful in the decision‐making process (Chan et al., 2018). Telephone counselling has been described as one suicide prevention strategy (Lapierre et al., 2011). However, with the exception of the study by Chan et al. (2018) in this review, most of the studies investigating the use of a telephone hotline were not specifically concerned with older adults. Nevertheless, it might be useful to investigate these studies and ascertain what was important in communication via a telecare helpline. However, Krysinska and De Leo (2007) revealed that a telecare helpline may put pressure on vulnerable individuals who are struggling with a suicide plan. Communication in a supportive and understanding environment where they can share their feelings and thoughts seems to be crucial for suicidal adults (Krysinska & De Leo, 2007). Nevertheless, mental health professionals have advised against such counselling online discussion groups and chat forums that focus on suicide (Krysinska & De Leo, 2007). Despite these concerns, it may be important to have a dialogue with older adults about suicide ideation to determine whether they have a suicide plan. Dialogue and communication should demonstrate a genuine concern about and understanding of older adults’ desire to be relieved of their intolerable emotional pain (Holm & Severinsson, 2015).

*Social support and awareness of causing significant others emotional pain* are important prevention strategies. Older adults can be so overwhelmed by their own emotional pain that they appear unaware of causing pain to others. Thus, in this way, the suicide risk can be related to social and environmental factors in addition to social attitudes. According to Pope (1976), Durkheim believed that suicide is a result of social and structural factors rather than individual ones. Such structural factors can include social ties with others, as well as the impact of societal rules and norms on individuals (Stanley et al., 2015). The systematic review by Holm and Severinsson (2015) supports the inclusion of social factors in the prevention of suicide in old age.

### 5.1 Limitations of an integrative review

Limitations of an integrative review can be related to the methodological quality of the empirical studies (Tables 1, Table S1) and the screening questions in the quantitative and qualitative studies. In this integrative review, no study was excluded because of the methodological quality. A rigorous literature review should consider including different issues (Hopia et al., 2016). One of the inclusion criteria was that the title of each study must include the words ‘suicide prevention strategies for older persons’ which could be a limitation in terms of the extent to which the review meets the appropriate standards for primary research (Evans, 2007). An audit trail was kept by the first author during the review process, as suggested by Miles and Huberman (1994) in order to document decisions, ideas, thoughts and issues that arose. Using several research methods in a review can be a challenge at the synthesis stage (Gough et al., 2019) and be a limitation of an integrative review. No consensus exist on methodological quality of an integrative review (Evans, 2007). This review has mainly used studies that has gone through a peer‐review process. A limitation might be that the search strategy excluded languages other than English. Bias can occur at any stage of a review process as suggested by Hopia et al. (2016). Other limitations can be related to culture, as the included empirical studies are from different parts of the world, for example five are from Asia (China, Singapore and South Korea), thus the authors might have a different perspective on suicide prevention strategies (Table 1). One empirical study was from Turkey, one was from France and one from the United States. As shown in Table 3, five of the theoretical studies are from the United States and one is from Turkey.

**TABLE 3:** Analysis and key aspects of the included quantitative (*n* = 6), qualitative (*n* = 2) and theoretical studies (*n* = 6)

	Author	Aim	Results	Conclusions	Key aspects that contribute to the themes in the empirical studies (*n* = 8), and the theoretical studies (*n* = 6)*
	Quantitative studies (*n* = 6)				Key aspects in the from which the themes were developed
1	Chan et al. (2011), HONG KONG	To compare 2‐year completed suicide and treatment rates in a pre‐intervention group enrolled in a regional elderly suicide prevention programme (ESPP) that adopted a two‐tiered multifaceted care management model and to examine the suicide rate trend in older adults in the pre‐ and postintervention.	The 2‐year completed suicide rate was 7.58% in the pre‐intervention group and 1.99% in the ESPP group. There was no difference in re‐attempt rates. At a population level, the suicide rate only dropped significantly in women aged 85 years and older, relative to the pre‐intervention period. A typical referral pathway and aftercare comprised a gatekeeper in the first tier (primary care physicians, social workers, frontline healthcare workers and elderly service volunteers and non‐psychiatric specialists in tertiary care) who identified an at risk case and made an urgent referral.	An elderly suicide prevention programme was associated with a reduced rate of completed suicide in older suicide attempters.	Theme 1 Recognizing older adults’ physical and/or mental health problems and referring them for help and treatment. Sub‐theme Training gatekeeper or para professionals
2	Chan et al. (2018), HONG KONG	To explore the potential usefulness and relevance of a telephone helpline service.	The users’ risk of suicide was greater at the early period of using the telephone helpline service. Men living alone and having a history of mental illness were also associated with increased risk. Based on the identified factors, an estimation system was developed with a sensitivity of 0.73 and specificity of 0.54.	By identifying a suicide risk profile and a distinct telephone calling pattern among the users, early detection and a warning system can be implemented to allow timely intervention to reduce the number of older adult suicides in the community.	Theme 1 Recognizing older adults’ physical and/or mental health problems and referring them for help and treatment. Theme 3 Communication and dialogue about warning signs.
3	Chauliac et al. (2016), FRANCE	To assess the impact of training gatekeepers in nursing homes for the management of suicidal crisis (SC) in residents. ‘Trained’ nursing homes and ‘untrained’ nursing homes were compared in terms of routine suicide prevention measures implemented at an institutional level and the identification of suicidal individuals.	The two nursing home groups did not present significantly different characteristics. In the nursing homes with trained staff, staff members were deemed better prepared to approach suicidal individuals. The detection of suicidal residents relied more on the whole staff and less on the psychologist alone when compared to nursing homes without trained staff. A significantly larger number of measures were taken to manage suicidal residents in the trained nursing homes. Suicidal residents were more frequently referred to the psychologist.	Having trained gatekeepers has an impact not only for the trained individuals but also for the whole institution where they work, both in terms of managing suicidal residents and routine suicide prevention measures.	Theme 1 Recognizing older adults’ physical and/or mental health problems and referring them for help and treatment.
4	Ho et al. (2014), SINGAPORE	To examine the impact of a history of suicidal behaviour on suicide among elderly people in Singapore.	Elderly people who died by suicide and had a past history of suicidal behaviour were more likely to have suffered from major psychiatric disorders, encountered social problems in life, have alcohol, received psychiatric treatment in the past, were using anti‐depressive medication and were admitted to a mental hospital under the mental health legislation. Conversely, those without a past history of suicidal behaviour were more likely to have a pre‐suicidal plan and have received medical or surgical treatment in the past. For suicide prevention in Asians, psychiatrists should aggressively treat major psychiatric disorder, engage social services to resolve social problems in elderly older adults with a history of suicidal behaviour and reduce access to alcohol. Clinicians working in medical or surgical departments should routinely screen for suicide plans in elderly patients without a past history of suicidal behaviour.	For suicide prevention in Asians, psychiatrists should aggressively treat major psychiatric disorders, engage social services to resolve social problems in older adults with a history of suicidal behaviour, and reduce access to alcohol. Clinicians working in medical or surgical departments should routinely screen for suicide plans in older adults without a past history of suicidal behaviour.	Theme 1 Recognizing older adults’ physical and/or mental health problems and referring them for help and treatment. Theme 4 Need of social support and awareness of the emotional pain caused to significant others.
5	Kim& Yang (2017), SOUTH KOREA	To develop a small‐group focused suicide prevention programme for elders with early‐stage dementia and to assess its effects.	The suicide prevention programme had a significant effect on the perceived health status, social support, depression and suicidal ideation of elders with early‐stage dementia.	Nurses should integrate risk factors such as depression and protective factors such as health status and social support in a suicide prevention programme.	Theme 1 Recognizing older adults’ physical and/or mental health problems and referring them for help and treatment. Theme 4 Need of social support and awareness of the emotional pain caused to significant others.

	Qualitative studies (*n* = 2)				Key aspects in the qualitative studies from which the themes were developed
1	Vannoy et al. (2018), USA	To describe: a) what primary care providers can do to prevent late‐life suicide and b) older men's attitudes towards discussing suicide with a primary care provider.	Several themes that can prevent suicide emerged: It is a problematic solution, due to religious prohibition, conflicts with self‐image, the impact on others and lack of means/capacity. Three approaches to preventing suicide emerged: talking with the older men about depression, talking about the impact of their suicide on others and encouraging them to be active. 99% were open to such conversations. An unexpected theme spontaneously arouse: ‘What prevents men from acting on suicidal thoughts’?	Suicide is rarely discussed in primary care encounters in the context of depression treatment. This study suggested that older men are likely to be open to discussing suicide with their primary care professional.	Theme 1 Recognizing older adults’ physical and/or mental health problems and referring them for help and treatment. Theme 3 Communication and dialogue about warning signs Theme 4 Need of social support and awareness of the emotional pain caused to significant others.
2	Wong et al. (2011), CHINA	To examine the risk factors for suicide in elderly HONG KONG Chinese.	8 patients in this programme committed suicide during this period, 65% were male. All the suicides ensued within the first 6 weeks of treatment. These patients were diagnosed with depression and had an associated physical illness.	The first 2 months of treatment were associated with the highest risk of suicide. Intensive care and support including education about the effects of antidepressants and building a trusting therapeutic alliance with close relatives are particularly important during this vulnerable period.	Theme 1 Recognizing older adults’ physical and/or mental health problems and referring them for help and treatment. Theme 2 Designing an educational programme Theme 4 Need of social support and awareness of the emotional pain caused to significant others.

	Theoretical studies (*n* = 6)				Key aspects in the studies from which the themes were developed
	Arbore (2019), USA	What can we do?	Rural‐specific factors include shortages of trained professionals in eldercare services, difficulty accessing care, lack of transportation and stigma associated with seeking mental health treatment. Paraprofessionals may be a path forward.	We must identify older adults in rural areas who are experiencing emotional pain; we must respond to their needs; we must refer these individuals to people who can help them. Together we can make a difference, together we can save lives.	Theme 1 Recognizing older adults’ physical and/or mental health problems and referring them for help and treatment. Sub‐theme Training gatekeepers or para professionals. Theme 4 Need of social support and awareness of the emotional pain caused to significant others.
	Butcher and Ingram (2018), USA	To help healthcare providers to recognize those at risk of suicide and recommend appropriate and effective secondary suicide prevention interventions.	Crisis intervention to ensure safety is the highest level of secondary prevention. This includes hospitalization, increased surveillance, day treatment, antidepressant medications, communicating risk to family, appointment of care manager. Other interventions are Counselling, support groups, assistance to resolve family discord, assistance with financial stress, increasing social involvement, increasing activity with faith community.	In the wake of the increased media attention to suicide deaths, now is the time to make suicide prevention for older adults a global healthcare priority.	Theme 1 Recognizing older adults’ physical and/or mental health problems and referring them for help and treatment. Theme 3 Communication and dialogue about warning signs. Theme 4 Need of social support and awareness of the emotional pain caused to significant others.
	Conwell (2014), USA	To outline priorities for prevention research and programming at the individual, service system and community levels.	Prevention strategies that decrease suicide deaths in later life should emphasize four areas. 1) early detection of older people at risk through improved understanding of multidimensional determinants and their interactions, 2) general health promotion that optimizes well‐being and independent functioning, 3) mental health care that is evidence‐based, accessible, affordable, acceptable and integrated with other aspects of care and 4) improvement of social connectedness. Including gatekeeper training about suicide warning signs will mobilize a helpful response to suicide.	There is no doubt about the importance of continuing to study interventions that target older people at risk. The highly lethal nature of suicidal behaviour in later life indicates that studying more distal risk factors and approaches to their mitigation and prevention will be necessary if a substantial reduction in the number of older adults taking their own lives is to be achieved.	Theme 1 Recognizing older adults’ physical and/or mental health problems and referring them for help and treatment. Sub‐theme Training gatekeepers or para professionals Theme 4 Need of social support and awareness of the emotional pain caused to significant others.
	Demirin et al. (2011), TURKEY	To evaluate precautions along with the characteristics of a suicidal case within existing published work.	There is no way of knowing how many of the deceased had a history of repeated suicide attempts. It is suggested that this could be attributed to routine failure to observe and mitigate risk factors. The magnitude of this problem is demonstrated by the case report presented about a planned complex suicide with multiple sharp injuries and substance overdose. This completed suicide was potentially preventable with timely detection and intervention.	The case reported here is a first step in responding to the gaps and understanding the challenges and issues associated with suicidal risk factors in the older population. It is only by informing the entire workforce about the need to care for older people that we can address how to handle the increasing number of older members in society and best manage their needs.	Theme 1 Recognizing older adults’ physical and/or mental health problems and referring them for help and treatment. Theme 4 Need of social support and awareness of the emotional pain caused to significant others.
	Fullen (2016), USA	To introduce a community‐based suicide prevention strategy that employs health and mental health professionals, aging sector professionals, caregivers and older adults to recognize and respond to older adults who may be in distress.	A comprehensive framework for preventing older adult suicide should include knowledge about risk factors, a theoretical basis that explains the high prevalence of older adult suicide and a transdisciplinary prevention strategy that shares responsibility for suicide prevention among professional and lay members of the community. Including gatekeeper training about suicide warning signs will mobilize a helpful response to suicide.	The synthesis of numerous perspectives and paradigms strengthens the community‐based approach to suicide prevention. A comprehensive, multidisciplinary approach reconstructs the meaning that society places on the aging process, leading to a consistent message that older lives have a purpose. Employing various professionals and laypeople within the community increases the commitment of all involved and communicates to members of the community, including older adults, that preventing suicide is a responsibility shared by all.	Theme 1 Recognizing older adults’ physical and/or mental health problems and referring them for help and treatment. Sub‐theme Training gatekeepers or para professionals. Theme 2 Designing an educational programme. Theme 3 Communication and dialogue about warning signs.
	Van Orden and Deming (2018), USA	To address key research findings over the past several years as well as what these findings suggest about directions needed to prevent suicide in later life.	The findings indicate an association between internalized ageist stereotypes and reduced will to live. Recent research addresses the role of cognitive control as contributor to risk and as an intervention target as well as firearm safety as a promising, though a politicized and challenging strategy to implement. Geriatricians routinely target numerous late‐life suicide risk factors—physical illness, functioning, pain and dissatisfaction with life. Efficacious strategies will not prevent suicide deaths if they are not implemented—addressing ageism as a universal prevention strategy is essential.	Late‐life suicide prevention must be broad and reach into our communities. Our dialogue about aging must be changed to one that is not only more positive, but more realistic.	Theme 1 Recognizing older adults’ physical and/or mental health problems and referring them for help and treatment. Theme 3 Communication and dialogue about warning signs. Theme 4 Need of social support and awareness of the emotional pain caused to significant others.

* Key aspects: Theme 1: Recognizing older adults’ physical and/or mental health problems and referring them for help and treatment. Sub‐theme: Training gatekeepers or paraprofessionals. Theme 2: Designing an educational programme. Theme 3: Communication and dialogue about warning signs and Theme 4: Need of social support and awareness of the emotional pain caused to significant others.

### 5.2 Implication for clinical practice

Negative attitudes about ageing and health can have implications for practice and affect mental health, as well as cognitive and physical functioning, thus leading to the risk of suicide in older adults (Levy & McDonald, 2016). Suicide prevention strategies need to address such negative attitudes and ageism that are often a part of community culture (Levy & McDonald, 2016).

An important implication for clinical practice is early detection of older suicidal adults (Conwell, 2014). Healthcare professionals working with older adults must look for signs of existential depression that can be related to intense emotional pain and the sense that life is no longer worth living (Holm et al., 2014; Holm, Lyberg, Berggren, Cutcliffe, et al., 2014). A suicide prevention programme can be developed, including a combination of treatment for depression, gatekeeper training and aftercare (Chan et al., 2011). Collaborative care models including psychotherapies to reduce suicidal thinking can be important in clinical practice, especially for depressed older adults (Van Orden & Deming, 2018). Healthcare professionals in community mental health care can develop a day‐care programme with home visits and engage family support on weekends (Fullen, 2016). Family members in a caregiver role need support from professionals in order to recognize suicide risk factors and respond to a family member in distress (Fullen, 2016).

A peer‐based prevention strategy can function by observing the behaviours of one's peers through interactions in the community (Podgorski et al., 2010). Social isolation and loneliness are a serious risk factor and Van Orden and Deming (2018) suggested providing peer companionship to reduce loneliness and behavioural therapy to increase social engagement in order to enhance social communication in senior centres and senior living communities. Several community‐based programmes in nursing practice can be effective in preventing suicide among older adults with early‐stage dementia (Kim & Yang, 2017).

It is essential to educate students about how to identify, evaluate and manage this problem (Karakus et al., 2015). Thus, healthcare professionals must be aware of warning signs and communicate about the likelihood of the presence of a suicide risk (Chan et al., 2018). As found in this review, healthcare professionals must find time to ask questions about suicide plans.

### 5.3 Implications for research

Research could highlight different groups of older adults suffering from a mental health disorder or early‐stage dementia and examine family, neighbourhood and community‐level risks and protective factors (Conwell, 2014; Kim & Yang, 2017). Research can include primary care screening and design studies to make care systems more effective in detecting and treating those at risk (Conwell, 2014). Research at community level should also focus on gatekeeper training for all who may have access to older adults in trouble (Conwell, 2014). More studies are needed on how to improve care delivery. In addition, studies about how to educate patients and families about the benefits of treatment for mental disorders and ensure that treatments are tailored to patient‐ and family‐centred care are also required (Conwell, 2014). Depression is common in older adults, but intervention studies are lacking (Fässberg et al., 2012). Thus, it is important to detect and intervene if suicide prevention strategies are to be effective (Kim & Yang, 2017). Further longitudinal studies are needed in Asia (Ho et al., 2014). Testing and providing peer companionship to reduce loneliness can be important (Van Orden et al., 2013). Research into gatekeeper training can be a vital step in evaluating its effectiveness and illuminating whether gatekeeper training is relevant to participants and communities (Isaac et al., 2009).

## 6 CONCLUSION

The evidence from this review highlights important aspects of suicide prevention strategies for older adults. Being in communication and dialogue about warning signs can be a first step to reducing the large number of suicides among older adults around the world. Healthcare professionals should focus on the fact that suicide is a way of escaping intense emotional pain. In addition, suicide prevention strategies need to address and resolve social contact problems. Several interventions designed to increase social contact and connectedness have led to a reduction in deaths by suicide. Efforts to enhance the living conditions of older adults, such as the establishment of retirement communities, can provide increased support and social networks, thus preventing late‐life suicide.

## CONFLICT OF INTEREST

We confirm that this study did not include any conflict of interest.

## AUTHOR CONTRIBUTIONS

ALH: Study design and data collection. ALH, ES and ES: Data analysis, discussion and preparation.

## ETHICAL APPROVAL

There is no need to include any patient consent statement or ethical approval details in an integrative review.

## Supporting information

Supplementary Material

## Data Availability

The data used and analysed in this study are available from the corresponding author for reasonable requests.
