# Supplementary material for: Suicide prevention strategies for older persons—An integrative review of empirical and theoretical papers
Source: Nurs Open. 2021 Feb 23;8(5):2175–93. doi: 10.1002/nop2.789 (PMC8363358; doi:10.1002/nop2.789)
Supplement: Supplementary file 1 — Supplementary Material [file NOP2-8-2175-s001.doc]

**Supplementary file (S1)**  Methodological quality criteria (Mixed Methods Appraisal Tool [MMAT, version 2018])

| Qualitative | 1 | 2 | 3 | 4 | 5 |
| --- | --- | --- | --- | --- | --- |
| Vannoy et al. (2018) | Y | Y | Y | Y | Y |
| Wong et al. (2011) | Y | Y | U | U | U |

Qualitative;

1) Is the qualitative approach appropriate to answer the research question?

2) Are the qualitative data collection methods adequate to address the research question?

3) Are the findings adequately derived from the data?

4) Is the interpretation of results sufficiently substantiated by data?

5) Is there coherence between qualitative data sources, collection, analysis and interpretation?

| Screening questions (for all types) | 1 | 2 | 3 | 4 | 5 |
| --- | --- | --- | --- | --- | --- |
| Chan et al. (2011) | Y | Y | U | U | Y |
| Chan et al. (2018) | Y | Y | U | U | Y |
| Ho et al.  (2014) | Y | Y | U | Y | Y |
| Karakus et al. (2015) | Y | Y | U | U | U |
|  |  |  |  |  |  |

Quantitative non-randomized

1) Are the participants representative of the target population?

2) Are measurements appropriate regarding both the outcome and intervention (or exposure)?

3) Are there complete outcome data?

4) Are the confounders accounted for in the design and analysis?

5) During the study period, was the intervention administered (or did exposure occur as intended?

| Screening questions (for all types) | 1 | 2 | 3 | 4 | 5 |
| --- | --- | --- | --- | --- | --- |
| Chauliac et al.  (2016) | Y | Y | Y | U | Y |
| Kim & Yang (2017) | Y | Y | Y | U | Y |

Quantitative descriptive:

1) Is the sampling strategy relevant to address the research question?

2) Is the sample representative of the target population?

3) Are the measurements appropriate?

4) Is the risk of nonresponse bias low?

5) Is the statistical analysis appropriate to answer the research question?

Y=Yes, N=No, NI=Not identified, U=Uncertain
